# Supplementary material for: Glucose Tolerance Test and Pharmacokinetic Study of Kaempferia parviflora Extract in Healthy Subjects
Source: Nutrients. 2019 May 25;11(5):1176. doi: 10.3390/nu11051176 (PMC6566825; doi:10.3390/nu11051176)
Supplement: Supplementary file 1 [file nutrients-11-01176-s001.pdf]

Supplementary Tables

Table S1. General characteristics of participants.

Characteristics	Placebo (n = 15)	KP extract 90 mg/day (n = 15)	KP extract 180 mg/day (n = 14)	Total (n = 44)	*P*-value
Age (year)	26.9 ± 7.2	25.3 ± 5.1	28.3 ± 9.7	26.8 ± 7.4	0.932
Gender (male/female)	7/8	6/9	8/6	21/23	0.655
Body weight (kg)	54.3 ± 7.4	55.1 ± 7.8	57.0 ± 7.4	55.5 ± 7.4	0.452
Height (cm)	163.0 ± 7.5	162.8 ± 9.2	164.2 ± 6.0	163.2 ± 7.6	0.706
BMI (kg/m^2)	20.5 ± 2.3	20.8 ± 2.2	21.3 ± 2.2	20.8 ± 2.2	0.478
Systolic blood pressure (mmHg)	110 ± 15	113 ± 16	110 ± 14	111 ± 15	0.992
Diastolic blood pressure (mmHg)	69 ± 22	71 ± 9	67 ± 6	69 ± 14	0.423
Pulse rate (beats/min)	75 ± 9	77 ± 11	70 ± 13	74 ± 11	0.203
Body temperature (°C)	36 ± 3	36 ± 0	36 ± 0	36 ± 2	0.975

1

Table S2. Blood analysis of subjects at base line and at 28 days after KP treatment in glucose tolerance test.

**Parameters** (normal limits)	**Placebo (n = 15)**		**KP 90 mg/day (n = 15)**		**KP 180 mg/day (n = 14)**	
	**day 0**	**day 28**	**day 0**	**day 28**	**day 0**	**day 28**
RBC (3.9–5.6 x 10^6 µL)	5.2 ± 0.6	5.2 ± 0.7	5.4 ± 0.8	5.3 ± 0.8	5.4 ± 0.6	5.3 ± 0.5
WBC (3.8–10.8 x 10^3/µL)	5.9 ± 1.0	6.2 ± 1.5	6.6 ± 1.2	7.0 ± 1.3	7.0 ± 3.1	7.3 ± 2.6
Hb (12–18 g/dL)	13.0 ± 1.9	13.1 ± 1.9	12.7 ± 2.3	13.0 ± 1.5	14.1 ± 1.5	13.7 ± 1.3
Hct (37–42%)	40.3 ± 5.5	41 ± 5.6	40.2 ± 4.9	39.9 ± 4.9	43.0 ± 4.6	41.6 ± 3.7
Platelet count (140–400 x 10^3)	250 ± 59	246 ± 55	277 ± 69	282 ± 57	254 ± 40	226 ± 34
MCV (80–95 fL)	78.9 ± 11.1	80.2 ± 10.6	75.9 ± 12.9	77.5 ± 13.0	76.1 ± 14.3	80.4 ± 8.0

MCH<br><br>(27–32)	25.5 ± 3.4	25.8 ± 3.2	25.2 ± 3.9	25.2 ± 3.8	26.3 ± 2.6	26.1 ± 2.4
MCHC<br><br>(23–36)	32.5 ± 1.5	32.3 ± 1.5	33.4 ± 1.4	32.7 ± 1.4	33.2 ± 1.2	32.5 ± 0.8
RDW<br><br>(10.9–15.7)	14.8 ± 2.3	14.8 ± 2.1	14.5 ± 2.1	15.1 ± 2.4	14.1 ± 1.2	14.2 ± 1.0
Neutrophil<br><br>(44.3–70.9%)	50 ± 8	51 ± 8	53 ± 8	55 ± 7	50 ± 6	53 ± 9
Lymphocyte<br><br>(20.1–44.5%)	38 ± 7	38 ± 9	36 ± 8	34 ± 8	37 ± 6	35 ± 8
Monocyte<br><br>(3.4–9.8%)	6 ± 2	6 ± 1	7 ± 2	6 ± 1	7 ± 1	6 ± 2
Eosinophil<br><br>(0.7–9.2%)	6 ± 5	6 ± 5	4 ± 3	4 ± 2	6 ± 6	5 ± 5
Basophil<br><br>(0–2.6%)	0 ± 0	0 ± 0	0 ± 0	1 ± 1	1 ± 1	1 ± 0

Values are expressed as mean (95% CI)

Abbreviations: RBC, red blood cell count; WBC, white blood cell count; Hb, hemoglobin; Hct, hematocrit; Plt, platelet count; MCV, mean corpuscular volume; MCH, mean corpuscular hemoglobin; MCHC, mean corpuscular hemoglobin concentration; RDW, red blood cell distribution width

Table S3. Biochemical results of subjects at base line and at 28 days after KP treatment in glucose tolerance test.

**Parameters (normal limits)**	**Placebo (n = 15)**		**KP 90 mg/day (n = 15)**		**KP 180 mg/day (n = 14)**	
	**day 0**	**day 28**	**day 0**	**day 28**	**day 0**	**day 28**
Na (135–146 mEq/L)	140 ± 2	144 ± 3	140 ± 2	144 ± 3	139 ± 2	144 ± 2
K (3.5–5.5 mEq/L)	4.3 ± 0.2	4.0 ± 0.3	4.1 ± 0.3	3.9 ± 0.3	4.2 ± 0.3	4.0 ± 0.5
Cl (95–112 mEq/L)	104 ± 3	104 ± 2	104 ± 2	104 ± 2	102 ± 2	104 ± 2
CO_2 (22–32 mEq/L)	22.9 ± 1.6	19.0 ± 1.8	22.1 ± 1.9	18.7 ± 1.6	23.8 ± 1.9	19.8 ± 2.1
Mg (1.8–2.6 mg/dL)	2.2 ± 0.1	2.2 ± 0.2	2.2 ± 0.2	2.2 ± 0.2	2.3 ± 0.1	2.2 ± 0.1
Phosphate (2.5–4.5 mg/dL)	3.5 ± 0.5	4.0 ± 0.6	3.9 ± 0.4	4.1 ± 0.5	3.8 ± 0.5	4.0 ± 0.5

BUN (6–20 mg/dL)	13 ± 4	13 ± 2	13 ± 4	12 ± 4	14 ± 5	13 ± 4
Cr (0–1.5 mg/dL)	0.9 ± 0.3	0.9 ± 0.2	1.0 ± 0.3	0.9 ± 0.2	1.0 ± 0.3	0.9 ± 0.1
FPG (70–110 mg/dL)	87.1 ± 4.8	81.4 ± 20.8	87.7 ± 7.9	87.6 ± 6.1	87.0 ± 5.6	89.2 ± 10.2
AST (12–32 U/L)	19 ± 4	20 ± 4	18 ± 5	20 ± 11	22 ± 11	23 ± 18
ALT (4–36 U/L)	16 ± 6	18 ± 5	16 ± 6	18 ± 7	18 ± 9	19 ± 9
ALP (42–121 U/L)	65 ± 15	59 ± 14	62 ± 13	55 ± 11	59 ± 14	55 ± 15
Direct bilirubin (0–0.3 mg/dl)	0.1 ± 0.1	0.1 ± 0.1	0.1 ± 0.1	0.1 ± 0.1	0.1 ± 0.1	0.1 ± 0.1
Indirect bilirubin (0–1.2 mg/dl)	0.5 ± 0.2	0.4 ± 0.2	0.4 ± 0.3	0.4 ± 0.3	0.5 ± 0.2	0.3 ± 0.2

Total cholesterol<br><br>(<200 mg/dL)	189.1 ± 37.6	195.7 ± 34.4	194.3 ± 31.6	190.0 ± 21.6	198.2 ± 34.0	185.8 ± 35.5
Triglyceride<br><br>(<160 mg/dL)	78 ± 39	85 ± 64	70 ± 21	80 ± 30	78 ± 37	80 ± 30
LDL<br><br>(<100 mg/dL)	129 ± 28	133 ± 28	135 ± 37	124 ± 23	137 ± 30	120 ± 26
HDL<br><br>(40–60 mg/dL)	49 ± 14	45 ± 10	44 ± 11	50 ± 11	48 ± 10	50 ± 9

Values are expressed as mean (95% CI)

Abbreviations: Na, sodium; K, potassium; Cl, chloride; CO2, carbon dioxide; Mg, magnesium; BUN, blood urea nitrogen; Cr, creatinine; FPG, fasting plasma glucose; AST, aspartate aminotransferase; ALT, alanine aminotransferase; ALP, alkaline phosphatase; TC, total cholesterol; TG, triglyceride; LDL, low density lipoprotein; HDL, high density lipoprotein.
